# Doubly Bayesian Analysis of Confidence in Perceptual Decision-Making

**DOI:** 10.1371/journal.pcbi.1004519

**Published:** 2015-10-30

**Authors:** Laurence Aitchison, Dan Bang, Bahador Bahrami, Peter E. Latham

**Affiliations:** 1 Gatsby Computational Neuroscience Unit, University College London, London, United Kingdom; 2 Department of Experimental Psychology, University of Oxford, Oxford, United Kingdom; 3 Calleva Research Centre for Evolution and Human Sciences, Magdalen College, University of Oxford, Oxford, United Kingdom; 4 Interacting Minds Centre, Aarhus University, Aarhus, Denmark; 5 Institute of Cognitive Neuroscience, University College London, London, United Kingdom; Imperial College London, UNITED KINGDOM

## Abstract

Humans stand out from other animals in that they are able to explicitly report on the reliability of their internal operations. This ability, which is known as metacognition, is typically studied by asking people to report their confidence in the correctness of some decision. However, the computations underlying confidence reports remain unclear. In this paper, we present a fully Bayesian method for directly comparing models of confidence. Using a visual two-interval forced-choice task, we tested whether confidence reports reflect heuristic computations (e.g. the magnitude of sensory data) or Bayes optimal ones (i.e. how likely a decision is to be correct given the sensory data). In a standard design in which subjects were first asked to make a decision, and only then gave their confidence, subjects were mostly Bayes optimal. In contrast, in a less-commonly used design in which subjects indicated their confidence and decision simultaneously, they were roughly equally likely to use the Bayes optimal strategy or to use a heuristic but suboptimal strategy. Our results suggest that, while people’s confidence reports can reflect Bayes optimal computations, even a small unusual twist or additional element of complexity can prevent optimality.

## Author Summary

Confidence plays a key role in group interactions: when people express an opinion, they almost always communicate—either implicitly or explicitly—their confidence, and the degree of confidence has a strong effect on listeners. Understanding both how confidence is generated and how it is interpreted are therefore critical for understanding group interactions. Here we ask: how do people generate their confidence? A priori, they could use a heuristic strategy (e.g. their confidence could scale more or less with the magnitude of the sensory data) or what we take to be an optimal strategy (i.e. their confidence is a function of the probability that their opinion is correct). We found, using Bayesian model selection, that confidence reports reflect probability correct, at least in more standard experimental designs. If this result extends to other domains, it would provide a relatively simple interpretation of confidence, and thus greatly extend our understanding of group interactions.

## Introduction

Humans and other animals use estimates about the reliability of their sensory data to guide behaviour (e.g. [1–3]). For instance, a monkey will wait until its sensory data is deemed sufficiently reliable before taking a risky decision [3]. Humans can go further than other animals: they can explicitly communicate estimates of the reliability of their sensory data, by saying, for instance, “I’m sure”—an ability that is important for effective cooperation [4–6]. This ability to report on the reliability of our internal operations is known as “metacognition”, and is typically studied by asking people to report their confidence in the correctness of some decision [7]. However, the computations underlying confidence reports remain a matter of debate (see Box 1 in [6], for a brief overview). For instance, in an orientation-discrimination task, reports might—as a heuristic—reflect the perceived tilt of a bar. Alternatively, reports might reflect more sophisticated computations, like Bayesian inference about the probability that a decision is correct. An accurate understanding of confidence reports is important given their role in high-risk domains, such as financial investment (e.g. [8]), medical diagnosis (e.g. [9]), jury verdicts (e.g. [10]), and politics (e.g. [11]).

Here, we ask: how do people compute their confidence in a decision? We are particularly interested in whether confidence reports reflect heuristic or Bayes optimal computations. The latter would be consistent with a wide array of work showing that other aspects of perception and decision making are Bayes optimal [12]. However, as far as we know, whether confidence reports reflect Bayes optimal computations has not been directly tested. We use a standard psychophysical task in which subjects receive sensory data, make a decision based on this data, and report how confident they are that their decision is correct. Our goal is to determine how subjects transform sensory data into a confidence report. In essence, we are asking: if we use **x** to denote the sensory data (**x** can be multi-dimensional) and *c* to denote a confidence report, what is the mapping from **x** to *c*? Alternatively, what is the function *c*(**x**)?

To answer this question, we follow an approach inspired by signal detection theory [13]. We hypothesize that subjects compute a continuous decision variable, *z* ^D^(**x**), and compare this variable to a single threshold to generate a decision, *d*. Likewise, we hypothesize that subjects compute a continuous confidence variable, *z* ^C^(**x**; *d*), an internal representation of the evidence in favour of the chosen decision, *d*, and compare this variable to a set of thresholds to generate a level of confidence, *c* (the evidence in favour of one decision is different from the evidence in favour of the other decision, so the confidence variable must not only depend on the sensory evidence, **x**, but also the decision, *d*). Within this framework, a heuristic computation is a reasonable, but ultimately somewhat arbitrary, function of the sensory data. For instance, if the task is to choose the larger of two signals, *x*_1_ or *x*_2_, a heuristic confidence variable might be the difference between the two signals: $z_{\Delta }^{C}\text{C}\left(x;d=2\right)=x_{2}-x_{1}$ (the subscript Δ denotes difference). The Bayes optimal confidence variable, on the other hand, is the probability that a correct decision has been made: $z_{\text{B}}^{\text{C}}(x;d)=P(\text{correct}|x,d)$ (the subscript *B* denotes Bayesian).

The question of whether confidence reports reflect Bayes optimal (or simply Bayesian) computations has important implications for inter-personal communication. In particular, probabilities, as generated by Bayes optimal computations, can easily be compared across different tasks (e.g. perception versus general knowledge), making them easier to map onto reports. In contrast, heuristic computations typically lead to task-dependent internal representations, with ranges and distributions that depend strongly on the task, making it difficult to map them onto reports consistently, or compare them between different people.

**Fig 1:**
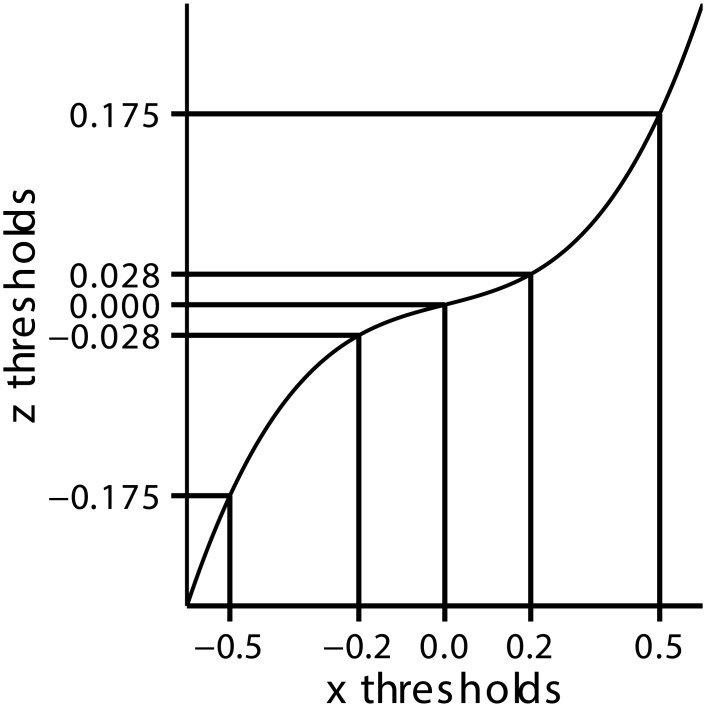
For one-dimensional sensory data, *x*, any monotonic transformation, *z*(*x*), can give the same mapping from *x* to *c*. The best we, as experimenters, can do is to determine the mapping from *x* to *c*, which, for discrete mappings, corresponds to a set of thresholds (the vertical lines). We can, however, get the same mapping from *x* to *c* by first transforming *x* to *z* (the curved black line), then thresholding *z*. The relevant thresholds are simply given by passing the *x*-thresholds through *z*(*x*) (giving the horizontal lines). Therefore, there is no way to determine the “right” *z*(*x*)—any *z*(*x*) will fit the data (as long as *z*(*x*) is a strictly monotonic function of *x*).

To our knowledge, it is impossible to determine directly the confidence variable, *z* ^C^(**x**; *d*); instead, we can consider several models, and ask which is most consistent with experimental data. Choosing among different models for the confidence variable, *z* ^C^(**x**; *d*), is straightforward in principle, but there are some subtleties. The most important subtlety is that if the task is “too simple”, it is impossible to distinguish one model from another. Here, “too simple” means that the sensory data, **x**, consists of a single signal, which we write *x* to indicate that it is scalar. To see why, let’s say we wanted to distinguish between some heuristic confidence variable, say $z_{\text{H}}^{\text{C}}\left(x;d\right)=x$, and the Bayes optimal confidence variable, $z_{\text{B}}^{\text{C}}\left(x;d\right)=P\left(\text{correct}|x,d\right)$. Suppose we found empirically that a subject reported low confidence when the heuristic variable, $z_{\text{H}}^{\text{C}}\left(x;d\right)$, was less than 0.3 and high confidence when the heuristic variable was greater than 0.3. Clearly there is a deterministic mapping from the heuristic variable to the confidence reports, but is it in any way unique? The answer is no. For example, if the Bayesian variable is greater than 0.4 whenever the heuristic variable is greater than 0.3, then it is also true that our subject reported low confidence when the Bayesian variable was less than 0.4 and high confidence when the Bayesian variable was greater than 0.4. Thus, there is absolutely no way of knowing whether our subjects’ confidence reports reflect the heuristic or the Bayesian confidence variable. In general, there is no way to distinguish between any two functions of *x* that are monotonically related—one can simply map the thresholds through the relevant function, as shown in Fig 1.

The situation is very different when **x** is a vector (i.e. two or more sensory signals). As in the one-dimensional case, consider two models: a heuristic model, $z_{\text{H}}^{\text{C}}\left(x;d\right)$, and a Bayes optimal model, $z_{\text{B}}^{\text{C}}\left(x;d\right)$. In general, if **x** is a vector, it is not possible to get the same mapping from **x** to *c* using $z_{\text{H}}^{\text{C}}\left(x;d\right)$ and $z_{\text{B}}^{\text{C}}\left(x;d\right)$. In particular, when $z_{\text{H}}^{\text{C}}\left(x;d\right)$ and $z_{\text{B}}^{\text{C}}\left(x;d\right)$ provide a different ordering of the **x**’s—whenever we have $z_{\text{H}}^{\text{C}}(x_{1};d)>z_{\text{H}}^{\text{C}}(x_{2};d)$ and simultaneously $z_{\text{B}}^{\text{C}}(x_{1};d)<z_{\text{B}}^{\text{C}}(x_{2};d)$—then it is not possible to find pairs of thresholds that lead to the same region in **x**-space. Thus, although we cannot say much about the confidence variable for one-dimensional signals, we can draw strong conclusions for multi-dimensional signals.

This difference between one-dimensional and multi-dimensional sensory data is one of the key differences between our work and most prior work. Previous models based on signal detection theory have typically assumed that the sensory data is one-dimensional (e.g. [14–16]), leaving them susceptible to the problem described above. There is also a variety of “dynamic” signal detection theory models in which sensory data is assumed to accumulate over time (see Pleskac & Busemeyer (2010) [17], for an overview). Such models are able to explain the interplay between accuracy, confidence, and reaction time—something that we leave for future work. However, in these models, the sensory data is also summarised by a single scalar value, making it impossible to determine whether subjects’ confidence reports reflect heuristic or Bayes optimal computations.

Here we considered multi-dimensional stimuli in a way that allows us to directly test whether subjects’ confidence reports reflect heuristic or Bayes optimal computations. In our study, subjects were asked to report their confidence in a visual two-interval forced-choice task. This allowed us to model the sensory data as having two dimensions, with one dimension coming from the first interval and the other from the second interval. We considered three models for how subjects generated their confidence—all three models were different “static” versions of the popular race model in which confidence reports are assumed to reflect the balance of evidence between two competing accumulators (originally proposed by Vickers (1979) [18], and more recently used in studies such as Kepecs *et al*. (2008) [1], and de Martino *et al*. (2013) [19]). The first model, the Difference model, assumed—in line with previous work—that subjects’ confidence reports reflected the difference in magnitude between the sensory data from each interval. The second model, the Max model, assumed that subjects’ confidence reports reflected only the magnitude of the sensory data from the interval selected on a given trial—thus implementing a “winner-take-all” dynamic [20]. The third model, the Bayes optimal model, assumed that subjects’ confidence reports reflected the probability that their decision was correct given the sensory data from each interval. Furthermore, we tested two different methods for eliciting confidence—both being used in research on metacognition [7]. In the standard two-response design, subjects first reported their decision, and only then, and on a separate scale, reported their confidence. In the less-commonly used one-response design, subjects reported their confidence and decision simultaneously on a single scale. We were interested to see whether the more complex one-response design—in which subjects, in effect, have to perform two tasks at the same time—affected the computations underlying confidence reports as expected under theories of cognitive load (e.g. [21, 22]) and dual-task interference (e.g. [23, 24]).

We used Bayesian model selection to assess how well the models fit our data; thus our analysis was “doubly Bayesian” in that we used Bayesian model selection to test whether our subjects’ behaviour was best explained by a Bayes optimal model [25]. We found that the commonly used Difference model was the least probable model irrespective of task design. Subjects’ confidence reports in the two-response design were far more likely to reflect the Bayes optimal model rather than either heuristic model. In contrast, in the one-response design, the confidence reports of roughly half of the subjects were in line with the Bayes optimal model, and the confidence reports of the other half were in line with the Max model, indicating that, perhaps, the increased cognitive load in the one-response paradigm caused subjects to behave suboptimally. In sum, our results indicate that while it is possible to generate confidence reports using Bayes optimal computations, it is not automatic—and can be promoted by certain types of task.

## Methods

### Participants

Participants were undergraduate and graduate students at the University of Oxford. 26 participants aged 18–30 took part in the study. All participants had normal or corrected-to-normal vision. The local ethics committee approved the study, and all participants provided written informed consent.

### Experimental details

#### Display parameters and response mode

Participants viewed an LED screen (ViewSonic VG2236wm-LED, resolution = 800 × 600) at a distance of 57 cm. The background luminance of the screen was 62.5 cd/m^2^. The screen was connected to a personal laptop (Toshiba Satellite Pro C660-29W) via a VGA splitter (Startech 2 Port VGA Video Splitter) and controlled by the Cogent toolbox (http://www.vislab.ucl.ac.uk/cogent.php/) for MATLAB (Mathworks Inc). Participants responded using a standard keyboard.

#### Design and procedure

Participants performed a two-interval forced-choice contrast discrimination task. On each trial, a central black fixation cross (width: 0.75 degrees of visual angle) appeared for a variable period, drawn uniformly from the range 500–1000 milliseconds. Two viewing intervals were then presented, separated by a blank display lasting 1000 milliseconds. Each interval lasted ∼ 83 milliseconds. In each interval, there were six vertically oriented Gabor patches (SD of the Gaussian envelope: 0.45 degrees of visual angle; spatial frequency: 1.5 cycles/degree of visual angle; baseline contrast: 0.10) organised around an imaginary circle (radius: 8 degrees of visual angle) at equal distances from each other.

In either the first or the second interval, one of the six Gabor patches (the visual target) had a slightly higher level of contrast than the others. The interval and location of the visual target were randomized across trials. The visual target was produced by adding one of 4 possible values (0.015, 0.035, 0.07, 0.15) to the baseline contrast (0.10) of the respective Gabor patch.

After the second interval there was a blank display, which lasted 500 milliseconds, and a response display. The response display prompted participants to indicate which interval they thought contained the visual target and how confident they felt about their decision. Participants were split into two groups. Each group performed a slightly different version of the task. The difference lay only in how decisions and confidence were indicated; the stimuli seen by the two groups were identical.

**Fig 2:**
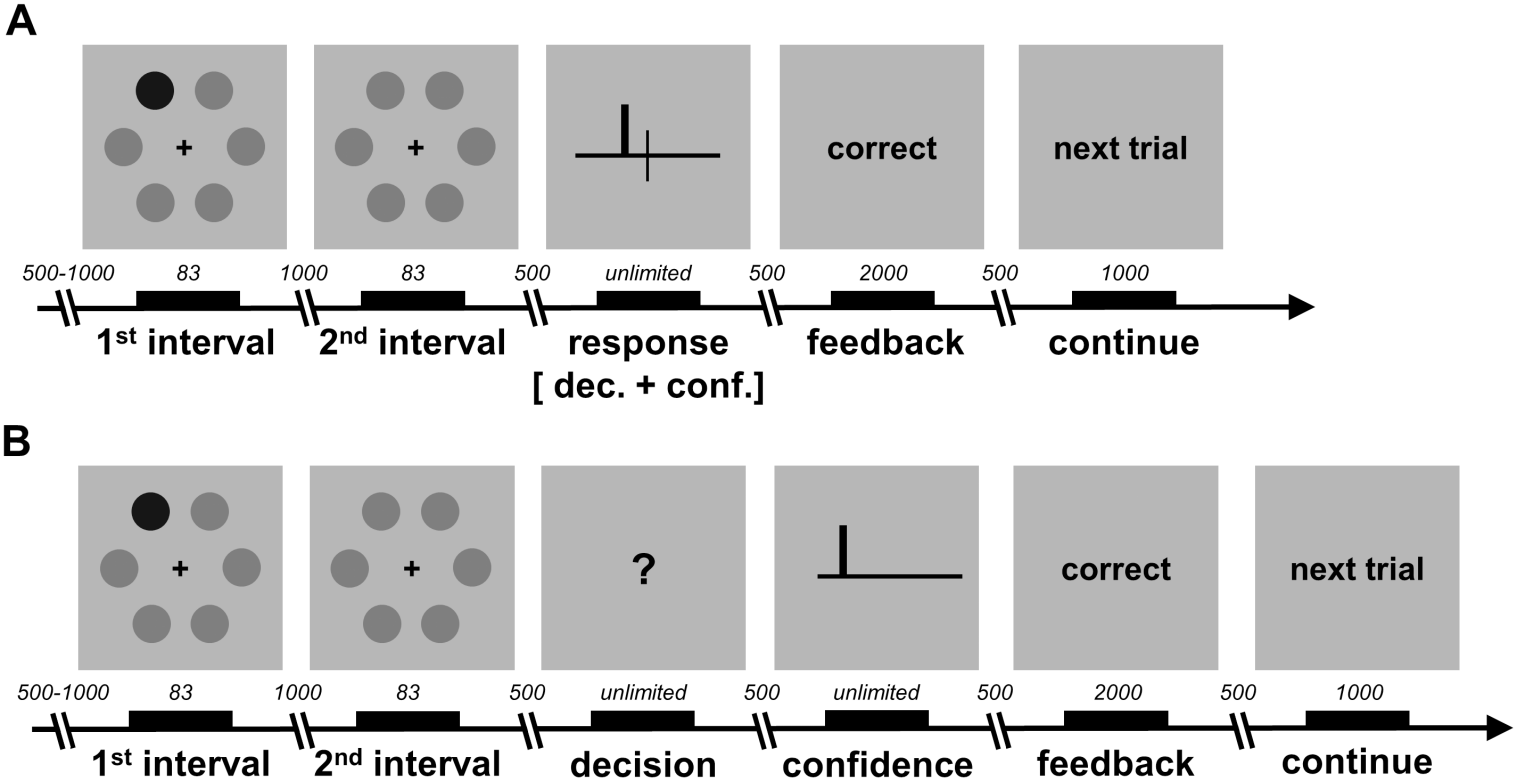
Schematic of experimental design and task. **A** One-response design. Participants indicated their decision and their confidence simultaneously. **B** Two-response design. Participants indicated their decision and their confidence sequentially. The displays have been edited for ease of illustration (e.g. Gabor patches are shown as dots, with the visual target being the darker dot). All timings are shown in milliseconds. See text for details.

For the first group, which had 15 participants, the response display consisted of a central black horizontal line with a fixed midpoint (Fig 2A). The region to the left of the midpoint represented the first interval; the region to the right represented the second interval. A vertical white marker was displayed on top of the midpoint. Participants were asked to indicate which interval they thought contained the visual target by moving the vertical marker to the left (first interval) or to the right (second interval) of the midpoint. The marker could be moved along the line by up to six steps on either side, with each step indicating higher confidence (1: “uncertain”; 6: “certain”). Participants pressed “N” or “M” to move the marker left or right, respectively, and locked the marker by pressing “B”.

For the second group, which had 11 participants, initially the response display consisted of a central black question mark (Fig 2B). Participants indicated which interval they thought contained the visual target, pressing “N” for the first interval and “M” for the second interval. After having indicated their decision, the response display switched to a central black horizontal line. A vertical white marker was displayed at the left extreme of the horizontal line. Participants indicated how confident they felt about their decision by moving the vertical marker along the line by up to six steps, with each step towards the right indicating higher confidence (1: “uncertain”; 6: “certain”). Participants pressed “N” or “M” to move the marker left or right, respectively, and locked the marker by pressing “B”.

After having made their response(s), participants were presented with central black text with either “correct” if their decision about the target interval was correct or “wrong” if it it was incorrect. The feedback display lasted 2000 milliseconds. Participants were then presented with central white text saying “next trial” before continuing to the next trial. Participants completed 16 practice trials followed by 480 experimental trials. The two groups were analysed separately. We refer to the two groups as “one-response” and “two-response”, respectively.

### Confidence models

To model responses, we assumed the following: On each trial, subjects receive a pair of sensory signals, **x**. Subjects transform those sensory signals into a continuous decision variable, *z* ^D^(**x**), and then compare this variable to a single threshold to make a decision, *d*. Finally, subjects transform the sensory signals and the decision into a continuous confidence variable, *z* ^C^(**x**; *d*), and then compare this variable to a set of thresholds to obtain a confidence report, *c*. This section starts by describing our assumptions about the sensory signals, **x**, then moves on to the models for how subjects might compute their decision and confidence variables. Finally, we describe the Bayesian inference technique used to fit the parameters and find the most probable model.

#### Sensory signals

We assumed that subjects on each trial receive two sensory signals, **x** = (*x*_1_, *x*_2_), drawn from two different Gaussian distributions, with *x*_1_ giving information about interval 1 and *x*_2_ giving information about interval 2. If the target is in interval 1, then

$$P\left(x_{1}|s,i=1,\sigma \right)=N\left(x_{1};s,\sigma^{2}/2\right)$$ (1a)

$$P\left(x_{2}|s,i=1,\sigma \right)=N\left(x_{2};0,\sigma^{2}/2\right),$$ (1b)

whereas if the visual target is in interval 2, then

$$P\left(x_{1}|s,i=2,\sigma \right)=N\left(x_{1};0,\sigma^{2}/2\right)$$ (2a)

$$P\left(x_{2}|s,i=2,\sigma \right)=N\left(x_{2};s,\sigma^{2}/2\right).$$ (2b)

Here *s* specifies the contrast added to the visual target, *s* ∈ {0.015, 0.035, 0.07, 0.15} as described in Design and Procedure, *i* ∈ {1, 2} denotes the target interval, and *σ* characterizes the level of noise in the subject’s perceptual system. The variance of each sensory signal is *σ* ^2^/2, which means that the variance of *x*_2_ − *x*_1_ is *σ* ^2^ as commonly assumed by psychophysical models.

#### Decision and confidence variables

We considered three models for how subjects compute their decision variable, *z* ^D^(**x**), and their confidence variable, *z* ^C^(**x**; *d*). We refer to these models as the Difference model (Δ), the Max model (M), and the Bayesian model (B). The Difference model proposes that the decision and the confidence variable reflect the difference between the two sensory signals,

$$z_{\Delta }^{D}(x)=x_{2}-x_{1}$$ (3)

$$z_{\Delta }^{C}(x;d)=\{\begin{array}{lll} x_{1}-x_{2} & \text{for} & d=1\\ x_{2}-x_{1} & \text{for} & d=2\;. \end{array}$$ (4)

In the next section we discuss how the decision, d (which is 1 for interval 1 and 2 for interval 2) is made.

The Max model proposes that the decision variable reflects the difference between the two sensory signals and the confidence variable reflects only the sensory signal received from the selected interval,

$$z_{\text{M}}^{\text{D}}(x)=x_{2}-x_{1},$$ (5)

$$z_{\text{M}}^{\text{C}}(x;d)=x_{d}.$$ (6)

Finally, the Bayesian model proposes that the decision variable reflects the probability that interval 2 contained the visual target, and that the confidence variable reflects the probability that the decision about the target interval is correct,

$$z_{\text{B}}^{\text{D}}(x)=P\left(i=2|x_{1},x_{2},\sigma \right)$$ (7)

$$z_{\text{B}}^{\text{C}}(x;d)=P\left(i=d|x_{1},x_{2},\sigma \right),$$ (8)

where

$$P\left(i=d|x_{1},x_{2},\sigma \right)=\frac{\sum_{s}P\left(x_{1}|s,i=d,\sigma \right)P\left(x_{2}|s,i=d,\sigma \right)}{\sum_{s,i\prime }P\left(x_{1}|s,i=i\prime ,\sigma \right)P\left(x_{2}|s,i=i\prime ,\sigma \right)}.$$ (9)

To derive this expression, we used Bayes′ theorem and assumed that the two conditions have equal prior probability (P(*i* = 1) = P(*i* = 2) = 1/2). The three models make different predictions about how the sensory signals contribute to the confidence variable, *z* ^C^(**x**; *d*), and therefore give rise to different confidence reports.

#### Choosing decisions and confidence reports

To make a decision, the subject compares the decision variable to a single threshold, and chooses interval 2 if the variable is larger than the threshold, and interval 1 otherwise,

$$d(x)=\left\{ \begin{array}{l} \begin{array}{cc} 2 & \text{if }z^{\text{D}}(x)>\theta^{D} \end{array}\\ \begin{array}{cc} 1 & \text{otherwise.} \end{array} \end{array}\right.$$ (10)

Likewise, to choose a confidence level, the subject compares their confidence variable to a set of thresholds, and the confidence level is then determined by the pair of thresholds that the confidence variable lies between. More specifically, the mapping from a confidence variable, *z* ^C^(**x**; *d*), to a confidence report, *c*, is determined implicitly by,

$$\theta_{d,c-1}^{\text{C}}<z^{\text{C}}\left(x;d\right)\leq \theta_{d,c}^{\text{C}}.$$ (11)

Valid confidence values, *c*, run from 1 to 6; to ensure that the whole range of *z* ^C^(**x**; *d*) is covered, we set *θ*_*d*, 0_ = −∞ and *θ*_*d*, 6_ = +∞.

Finally, we assumed that with some small probability *b*, subjects lapsed—they made a random decision and chose a random confidence level. Inclusion of this so-called lapse rate accounts for trials in which subjects made an otherwise low-probability response; e.g. they chose the first interval when there was strong evidence for the second. Such trials are probably due to some error (e.g. motor error or confusion of the two intervals), and if we did not include a lapse rate to explain these trials, they could have a strong effect on model selection.

### Model comparison

We wish to compute the probability of the various models given our data. The required probability is, via Bayes’ theorem,

P(m|data)∝P(m)P(data|m) (12)

where *m* is either Δ (Difference model), M (Max model) or B (Bayesian model). The data from subject *l* consists of two experimenter-defined variables: the target intervals, **i**_*l*_, and the target contrasts, **s**_*l*_, and two subject-defined variables: the subject’s decisions, **d**_*l*_, and the subject’s confidence reports, **c**_*l*_. Here, the bold symbols denote a vector, listing the value of that variable on every trial; for instance the interval on the *k* ^th^ trial is *i*_*lk*_. We fit different parameters to every subject, so the full likelihood, *P*(data∣*m*), is given by a product of single-subject likelihoods,

$$P(\text{data}|m)=\underset{l}{\prod }P\left(d_{l},c_{l},i_{l},s_{l}|m\right).$$ (13)

Because **i**_*l*_ and **s**_*l*_ are independent of the model, *m*, we may write

$$P(\text{data}|m)\propto \underset{l}{\prod }P\left(d_{l},c_{l}|i_{l},s_{l},m\right).$$ (14)

To compute the single-subject likelihood we cannot simply choose one setting for the parameters, because the data does not pin down the exact value of the parameters. Instead we integrate over possible parameter settings,

$$P\left(d_{l},c_{l}|i_{l},s_{l},m\right)=\int P\left(d_{l},c_{l}|i_{l},s_{l},m,\theta_{l},\sigma_{l},b_{l}\right)P\left(\theta_{l}\right)P\left(\sigma_{l}\right)P\left(b_{l}\right)d\theta_{l}d\sigma_{l}db_{l},$$ (15)

where *θ*_*l*_ collects that subject’s decision and confidence thresholds. This integral is large if the best fitting parameters explain the data well (i.e. if *P*(**d**_*l*_,**c**_*l*_∣**i**_*l*_,**s**_*l*_, *m*, ***θ***_*l*_, *σ*_*l*_, *b*_*l*_) is large for the best fitting parameters), as one might expect. However, this integral also takes into account a second important factor, the robustness of the model. In particular, a good model is not overly sensitive to the exact settings of the parameters—so you can perturb the parameters away from the best values, and still fit the data reasonably well. This integral optimally combines these two contributions: how well the best fitting model explains the data, and the model’s robustness. For a single subject (dropping the subject index, *l*, for simplicity, but still fitting different parameters for each subject), the probability of **d** and **c** given that subject’s parameters is the product of terms from each trial,

$$\begin{array}{c} P_{}(d,c|i,s,m,\theta ,\sigma ,b)=\prod_{k}P_{}(d_{k},c_{k}|i_{k},s_{k},m,\theta ,\sigma ,b)\;, \end{array}$$ (16)

We therefore need to compute the probability of a subject making a decision, *d*_*k*_, and choosing a confidence level, *c*_*k*_, given the subject’s parameters, the target interval, *i*_*k*_, and target contrast, *s*_*k*_. We do this numerically, by sampling: given a set of parameters, ***θ***, *σ* and *b* we generate an **x** from either Eqs (1) or (2) (depending on whether *i*_*k*_ is 1 or 2). We compute *z* ^D^(**x**) from either Eqs (3), (5) or (7) (depending on the model), and threshold *z* ^D^(**x**) to get a decision, *d*. Next, we combine **x** and *d* to compute *z* ^C^(**x**; *d*) from either Eqs (4), (6) or (8) (again, depending on the model), and threshold *z* ^C^(**x**; *d*) to get a confidence report, *c*. We do this many times (10^5^ in our simulations); *P*(*d*_*k*_, *c*_*k*_∣*i*_*k*_, *s*_*k*_, *m*, ***θ***, *σ*, *b*) is proportion of times the above procedure yields *d* = *d*_*k*_ and *c* = *c*_*k*_.

To perform the integral in Eq (15), we must specify prior distributions over the parameters *σ*, *b* and ***θ***. While it is straightforward to write down sensible priors over two of these parameters, *σ* and *b*, it is much more difficult to write down a sensible prior for the thresholds, ***θ***. This difficulty arises because the thresholds depend on *z* ^D^(**x**) and *z* ^C^(**x**; *d*), which change drastically from model to model. To get around this difficulty, we reparametrise the thresholds, as described in the next section.

#### Representation of thresholds

We reparametrise the decision and confidence thresholds in essentially the same way, but it is helpful to start with the decision threshold, as it is simpler. We exploit the fact that for a given model, there is a one to one relationship between the threshold, *θ* ^*D*^, and the probability that the subject chooses interval 1,

$$\begin{array}{c} p_{d=1}\equiv P_{}(d=1|m,\theta ,\sigma ,b)=\int_{-\infty }^{\theta^{D}}P_{}(z_{}^{\text{D}}|m,\sigma ,b)dz_{}^{\text{D}}\;. \end{array}$$ (17)

Therefore, if we specify the threshold, we specify *p*_*d* = 1_. Importantly, the converse is also true: if we specify *p*_*d* = 1_, we specify the threshold. Thus, we can use *p*_*d* = 1_ to parametrise the threshold. To compute the threshold from *p*_*d* = 1_, we represent *P*(*z* ^D^∣*m*, *σ*, *b*) using samples of *z* ^D^, which we can compute as described at the end of the previous section. To find the threshold, we sweep across possible values for the threshold, until the right proportion of samples are below the threshold (*p*_*d* = 1_), and the right proportion of samples are above the threshold (*p*_*d* = 2_).

The situation is exactly the same for confidence reports: if we specify the thresholds, we specify the distribution over confidence reports, *p*_*c*∣*d*_,

$$\begin{array}{c} p_{c|d}\equiv P_{}(c|d,m,\theta ,\sigma ,b)=\int_{\theta_{d,c-1}}^{\theta_{d,c}}P_{}(z_{}^{\text{C}}|d,m,\sigma ,b)dz_{}^{\text{C}}\;. \end{array}$$ (18)

Combining decision and confidence thresholds, we obtain the joint distribution over decisions and confidence reports, **p**, whose elements are

$$\begin{array}{c} p_{d,c}\equiv P_{}(d,c|m,\theta ,\sigma ,b), \end{array}$$ (19)

Thus, specifying the confidence and decision threshold specifies the joint distribution over decisions and confidence reports, **p**. Importantly, the reverse is also true: specifying **p** specifies the confidence and decision thresholds.

**Fig 3:**
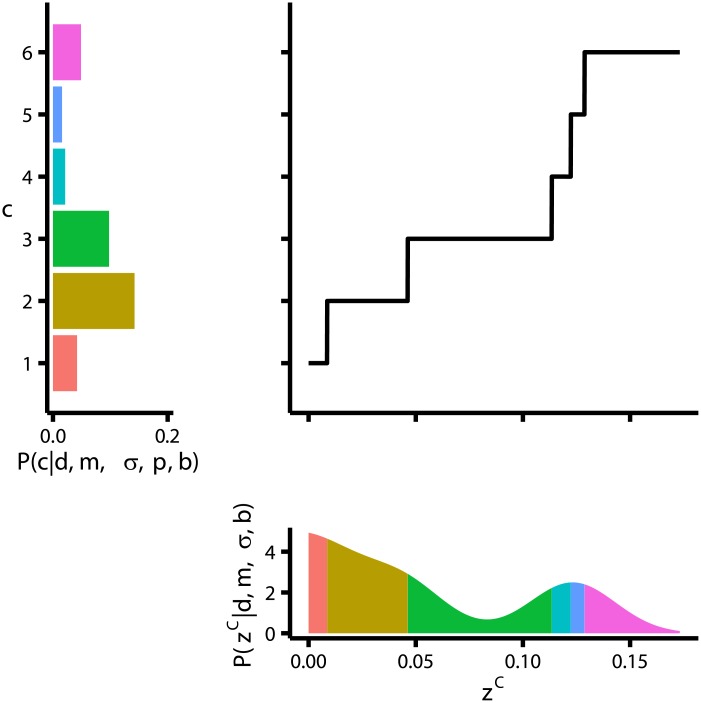
Schematic diagram of our method for mapping thresholds to confidence probabilities. The lower panel displays the (fixed) distribution over *z* ^C^, *P*(*z* ^C^∣*d*, *m*, *σ*, *b*) (which does not depend on the thresholds). The left panel displays the distribution over confidence reports, determined by **p**. The large central panel displays the fitted function mapping from *z* ^C^ to *c*, which consists of a set of jumps, with each jump corresponding to a threshold. The thresholds are chosen so that the total probability density in *P*(*z* ^C^∣*d*, *m*, *σ*, *b*) between jumps is exactly equal to the probability of the corresponding confidence level (see colours).

To find the confidence thresholds given **p**, we take the same strategy as for decisions—we represent *P*(*z* ^C^∣*d*, *m*, *σ*, *b*) using samples of *z* ^C^, then sweep across all possible values for the thresholds, until we get *c* = 1 the right fraction of the time (i.e. *p*_*c* = 1∣*d*_), and *c* = 2 the right fraction of the time (i.e. *p*_*c* = 2∣*d*_) etc. (see Fig 3 for a schematic diagram of this method). Note that, to condition on a particular decision, we simply throw away those values of *z* ^C^ associated with the wrong decision.

#### Performing the integral in Eq (15)

Changing the representation from thresholds, ***θ***, to probabilities, **p**, gives a new single-subject likelihood,

P(d,c|i,s,m)=∫P(d,c|i,s,m,p,σ,b)P(p)P(σ)P(b)dpdσdb. (20)

To perform the integral, we need to specify prior distributions over the parameters, *σ*, *b*, and **p**. For *σ*, we use

$$\begin{array}{c} P_{}(\sigma )=\text{Gamma}(2,0.05)\propto \sigma e^{-\sigma /0.05} \end{array}$$ (21)

as this broadly covered the range of plausible values of *σ*. We chose a very broad range of values for *b*—evenly distributed in log space between 10^−3^ and 10^−1^,

$$P\left({\text{log}}_{10}b\right)=\text{Uniform}\left(-3,-1\right).$$ (22)

Finally, we chose an uninformative, uniform prior distribution over **p**,

$$\begin{array}{c} P_{}(p)=\text{Dirichlet}(p;1), \end{array}$$ (23)

where **1** is a matrix whose elements are all 1.

The most straightforward way to compute the single-subject likelihood in Eq (20) is to find the average (expected) value of *P*(**d**,**c**∣**i**,**s**, *m*,**p**, *σ*, *b*) when we sample values of **p**, *σ* and *b* from the prior,

$$P(\text{data}|m)={\text{E}}_{P\left(p\right)P\left(\sigma \right)P\left(b\right)}[P(d,c|i,s,m,p,\sigma ,b)].$$ (24)

However, the likelihood, *P*(**d**,**c**∣**i**,**s**, *m*,**p**, *σ*, *b*), is very sharply peaked; being very high in a very small region around the subject’s true parameters, and very low elsewhere. The estimated value of the integral is therefore dominated by the few samples that are close to the true parameters, and as there are only a few such samples, the sample-based estimate of *P*(**d**,**c**∣**i**,**s**, *m*,**p**, *σ*, *b*) has high variance.

Instead, we use a technique called importance sampling. The aim is to find an equivalent expectation, in which the quantity to be averaged does not vary much, allowing the distribution to be estimated using a smaller number of samples—in fact, if the term inside the expectation is constant, then the expectation can be estimated using only one sample. Importance sampling uses

$$P(d,c|i,s,m)={\text{E}}_{Q\left(p\right)P\left(\sigma \right)P\left(b\right)}\left[\frac{P(d,c|i,s,m,p,\sigma ,b)P\left(p\right)}{Q\left(p\right)}\right].$$ (25)

The integral form for this expectation is,

$$P(d,c|i,s,m)=\int \frac{P(d,c|i,s,p,\sigma ,b)P\left(p\right)}{Q\left(p\right)}\;Q\left(p\right)P\left(\sigma \right)P\left(b\right)dpd\sigma db,$$ (26)

which is trivially equal to Eq (20). To ensure that the term inside the expectation in Eq (25) does not vary much, we need to choose the denominator, *Q*(**p**), so it is approximately proportional to the numerator, *P*(**d**,**c**∣**i**,**s**, *m*,**p**, *σ*, *b*)*P*(**p**). To do so, we exploit the fact that the numerator is proportional to a posterior distribution over **p** (considering only dependence on **p**),

P(d,c|i,s,m,p,σ,b)P(p)∝P(p|d,c,i,s,m,σ,b). (27)

Remembering that *p*_*d*, *c*_ is just the probability of a particular decision and confidence value, aggregating across all trial types, it is straightforward to construct a good approximation to the posterior over **p**. In particular, we ignore the influence of **i**, **s**, *m*, *σ* and *b*, so the only remaining information is the decisions and the confidence reports, **d** and **c**, irrespective of trial-type. These variables can be summarised by **n**, where *n*_*d*, *c*_, is the number of times that a subject chose decision *d* and confidence level *c*. The resulting distribution over **p** can be written,

Q(p)=P(p|d,c)=Dirichlet(p;1+n), (28)

which turns out to be a good proposal distribution for our importance sampler.

## Results

### Model selection

**Fig 4:**
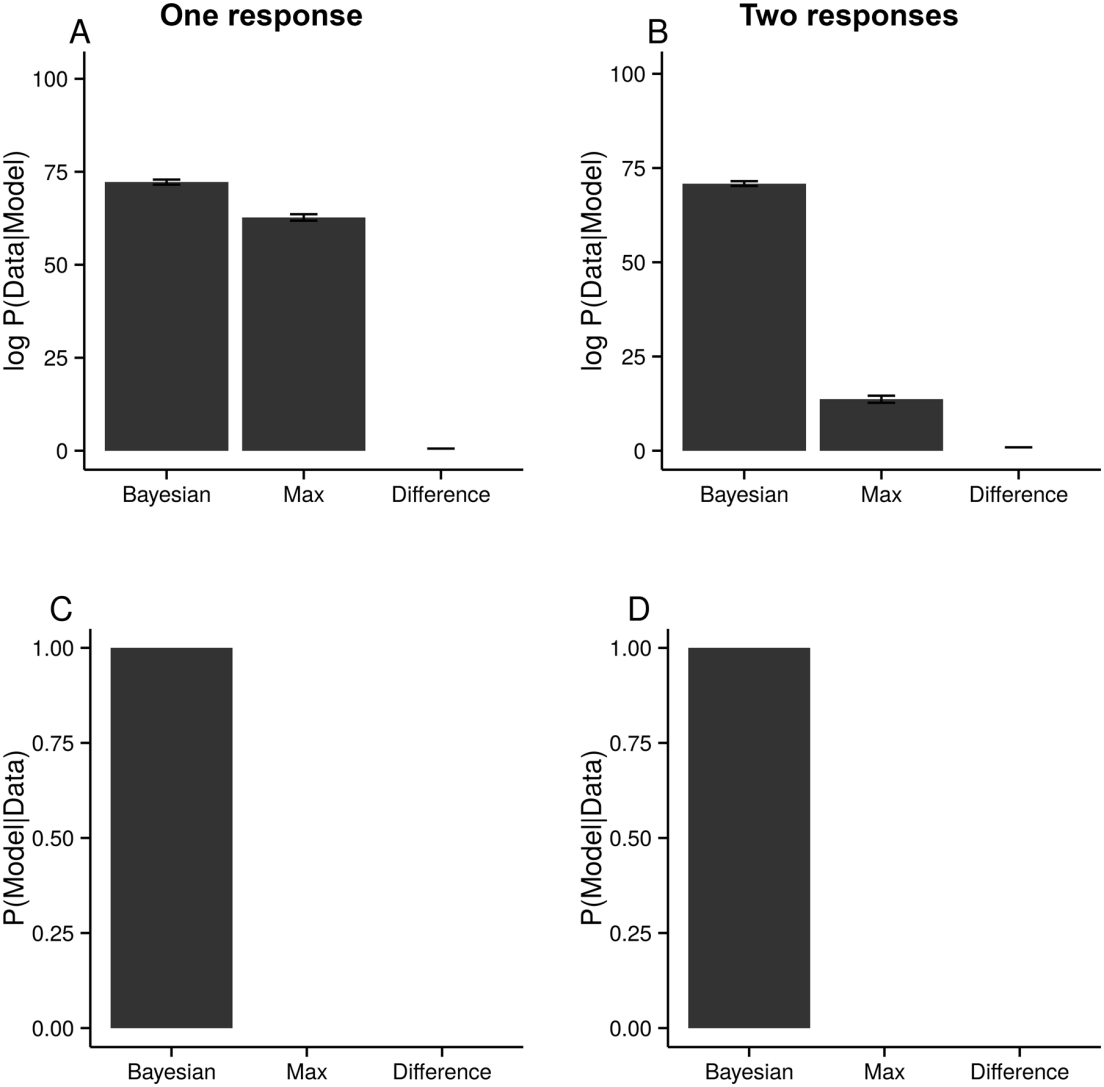
The probability of the three models given the data. **AB** The log-likelihood differences between the models, using the Difference model as a baseline. Note the small error bars, representing two standard-errors, given by running the algorithm 10 times, and each time using 1000 samples to estimate the model evidence (Eq (25)). **CD** The posterior probability of the models, assuming a uniform prior. Left column, one response. Right column, two responses.

To compare models, we look at the posterior probability of each of our models given the data, *P*(*m*∣data). As, a-priori, we have no reason to prefer one model over another, we use a uniform prior, *P*(*m*) = 1/3, so, assuming that every subject uses the same model, then the posterior is proportional to *P*(data∣*m*), which we showed how to compute in the Model Comparison Section. The Bayesian model is better by a factor of around 10^4^ for the one-response data and around 10^25^ for the two-response data (Fig 4).

**Fig 5:**
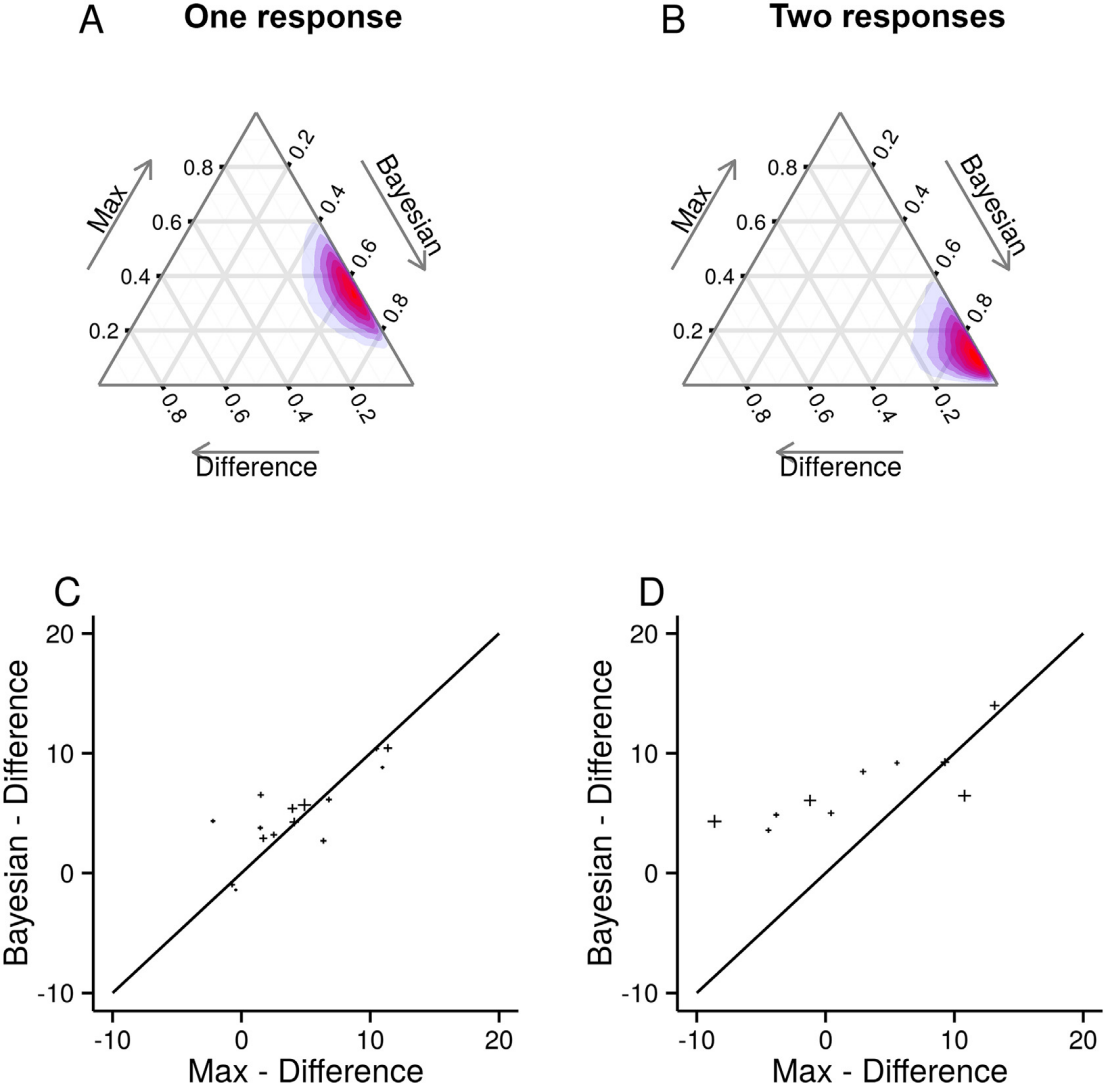
Single-subject analysis. **AB** Subjects are assumed to use each model with some probability. The coloured regions represent plausible settings for these probabilities. For the one-response dataset, we see that subjects are roughly equally likely to use the Max and Bayesian models. For the two-responses dataset, we see that subjects are far more likely to use the Bayesian model. To read these plots, follow the grid lines in the same direction as axis ticks and labels, so for instance, lines of equal probability for the Max model run horizontally, and lines of equal probability for the Bayesian model run up and to the right. **CD** The difference in log-likelihood between the Bayesian model and the Difference model (on the y-axis) against the difference in log-likelihood between the Max model and Difference model (on the x-axis). The size of the crosses represents the uncertainty (two standard errors) along each axis (based on the 10 runs of the model selection procedure, mentioned in Fig 4).

For the above model comparison, we assumed that all subjects used the same model to generate their confidence reports. It is quite possible, however, that different subjects use different models to generate their confidence reports. In particular, we might expect that there is some probability with which a random subject uses each model, *P*(*m*_*l*_) (where *l* is the subject index, so *m*_*l*_ is the model chosen by subject *l*). Under this assumption, we can analyse how well the models fit the data by inferring the probability with which subjects choose to use each model, *P*(*m*_*l*_), using a variational Bayesian method presented by [26]. In agreement with the previous analysis, we find that for the two-response dataset, the probability of any subject using the Bayesian model is high: subjects are significantly more likely to use the Bayesian model than either the Max or Difference models (*p* < 0.006; exceedence probability [26]; Fig 5B). For the one-response dataset, on the other hand, subjects use the Bayesian model only slightly more than the Max model (Fig 5A). The log-likelihood differences for individual subjects are plotted in Fig 5C and 5D, with uncertainty given by the size of the crosses. Again, for the two-response dataset, but not for the one-response dataset, the difference between each subject’s log-likelihood for the Bayesian and Max models is larger than 0 (two-response: *t*(10) = 3.47, *p* < .006; one-response: *t*(14) = 0.954, *p* ≈ .35; two-sided one-sample *t*-test).

### Model fits

**Fig 6:**
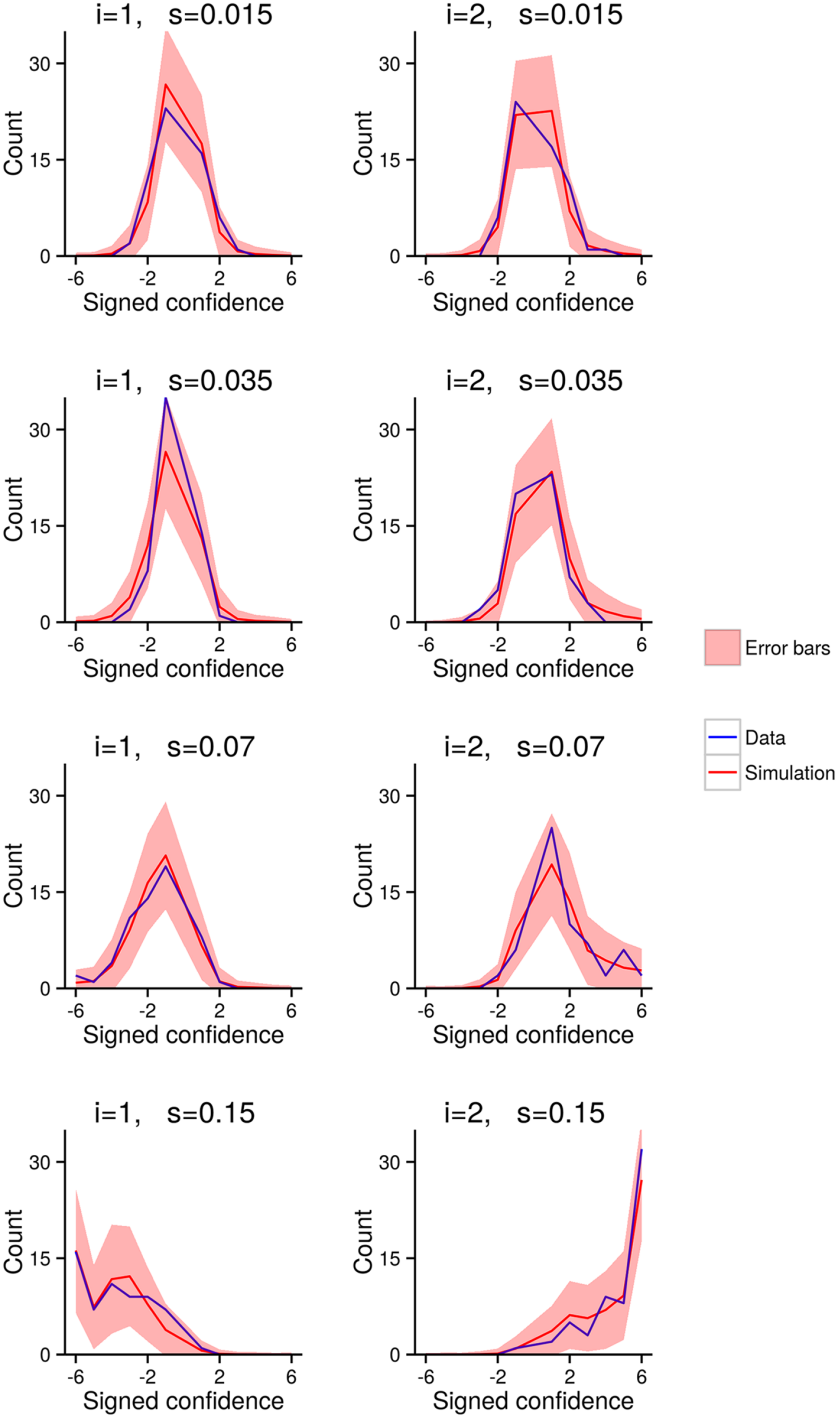
Simulated (Bayesian model) and actual confidence distributions for one subject (one response), and each target interval and contrast. The plots on the left are for targets in interval 1 (i.e. *i* = 1), whereas the plots on the right are for targets in interval 2 (i.e. *i* = 2). We use signed confidence on the horizontal axis (the sign indicates the decision, and the absolute value indicates the confidence level). The blue line is the empirically measured confidence distribution. The red line is Bayesian model’s fitted confidence distribution. The red area is the region around the fitted mean confidence distribution that we expect the data to lie within. We computed the error bars by sampling settings for the model parameters, then sampling datasets conditioned on those parameters. The error bars represent two standard deviations of those samples. This plot demonstrates that the Bayesian model is, at least, plausible.

While the model evidence is the right way to compare models, it is important to check that the inferred models and parameter settings (for inferred parameters for each subject see S1 and S2 Tables) are plausible. We therefore plotted the raw data—the number of times a participant reported a particular decision and confidence level for a particular target interval and target contrast—along with the predictions from the Bayesian model. In particular, in Fig 6, we plot fitted and empirical distributions over confidence reports given a target interval and contrast from an example participant (for all subjects and all models see S1 and S2 Figs). To make this comparison, we defined “signed confidence”, whose absolute value gives the confidence level, and whose sign gives the decision,

Signedconfidence={-cford=1-cford=2. (29)

**Fig 7:**
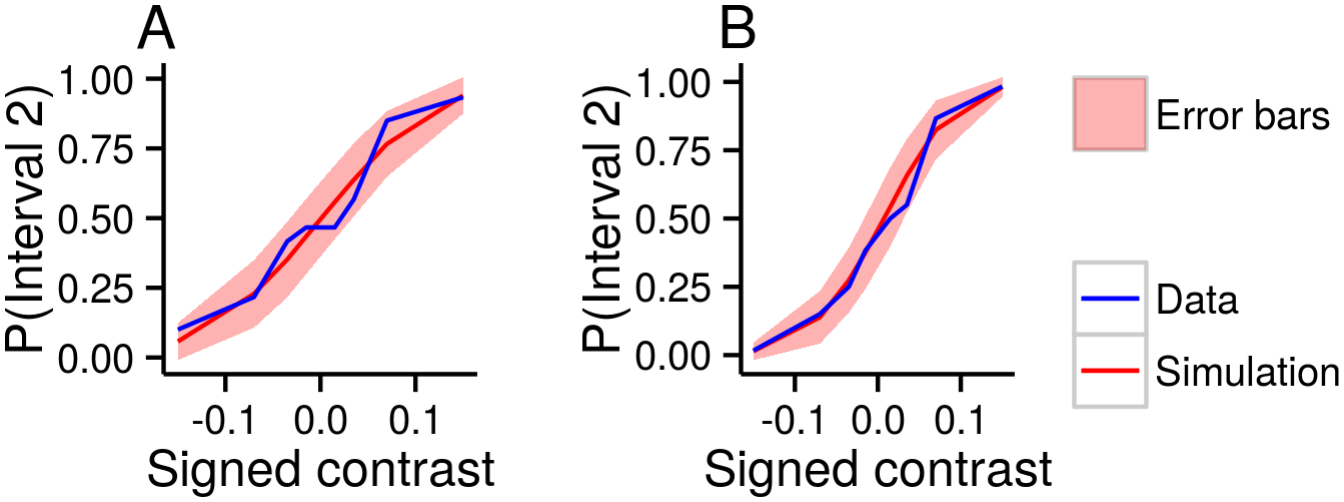
Simulated (Bayesian model) and actual psychometric curves for two subjects. The horizontal axis displays signed contrast (the sign gives the target interval, and the absolute value gives the contrast level). Colour code is the same as in Fig 6: the blue line is the empirically measured psychometric curve; the red line is the Bayesian model’s fitted mean psychometric curve; and the red area represents Bayesian error bars. **A** One subject from the one-response design. **B** One subject from the two-response design. As with Fig 6, this plot demonstrates the plausibility of the Bayesian model.

These plots show that our model is, at least, plausible, and highlights the fact that our model selection procedure is able to find extremely subtle differences between models. Plotting psychometric curves (Fig 7) gave similar results. Again, to plot psychometric curves, we defined “signed contrast”, whose absolute value gives the contrast, and whose sign gives the target interval,

Signedcontrast={-sfori=1-sfori=2. (30)

### Differences between models

For model selection to actually work, there need to be differences between the predictions made by the three models. Here, we show that the models do indeed make different predictions under representative settings for the parameters.

**Fig 8:**
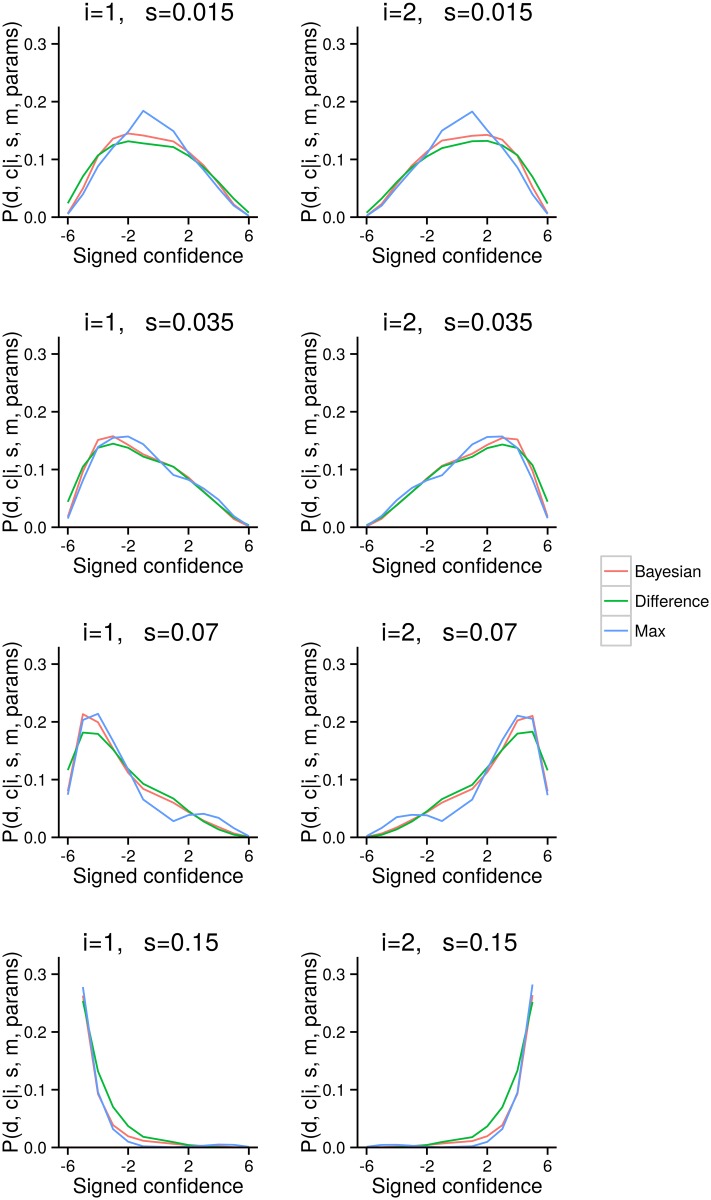
Different models lead to different distributions over confidence. Same as Fig 6, but displaying theoretical distributions induced by the three different models. The parameters were not fit to data; instead, they were set to fixed (but reasonable) values: *σ* = 0.07, *b* = 0 and *p*_*d*, *c*_ = 1/12.

To understand which predictions are most relevant, we have to think about exactly what form our data takes. In our experiment, we present subjects with a target in one of the two intervals, *i*, with one of four contrast levels, *s*, then observe their decision, *d* and confidence report, *c*. Overall, we therefore obtain an empirical estimate of each subject’s distribution over decision and confidence reports (or equivalently signed confidence, see previous section), given a target interval and contrast. This suggests that we should examine the predictions that each model makes about each subject’s distribution over decisions and confidence reports, given the target interval, *i*, and contrast, *s*. While these distributions are superficially very similar (Fig 8), closer examination reveals two interesting, albeit small, differences. Importantly, these plots display theoretical, and hence noise-free results, so even small differences are meaningful, and are not fluctuations due to noise.

First, the Max model differs from the other two models at intermediate contrast levels, especially *s* = 0.07, where the Max model displays bimodality in the confidence distribution. In particular, and unexpectedly, an error with confidence level 1 is less likely than an error with confidence levels 2 to 4. In contrast, the other models display smooth, unimodal behaviour across the different confidence levels. This pattern arises because the Max model uses only one of the two sensory signals. For example, when *s* = 0.07 and *i* = 2 (so the target is fairly easy to see, and is in interval 2), then *x*_2_ is usually large. Therefore, for *x*_1_ to be larger than *x*_2_, prompting an error, *x*_1_ must also be large. Under the Max model, *x*_1_ being large implies high confidence, and, in this case, a high confidence error.

Second, the three models exist on a continuum, with the Max model using the narrowest range of confidence levels, the Bayesian model using an intermediate range, and the Difference model using the broadest range. These trends are particularly evident at the lowest and highest contrast levels. At the lowest contrast level, *s* = 0.015, the distribution for the Max model is more peaked, whereas the distribution for the Difference model is lower and broader, and the Bayesian model lies somewhere between them. At the highest contrast level, *s* = 0.15, the Max model decays most rapidly, followed by the Bayesian model, and then the Difference model.

**Fig 9:**
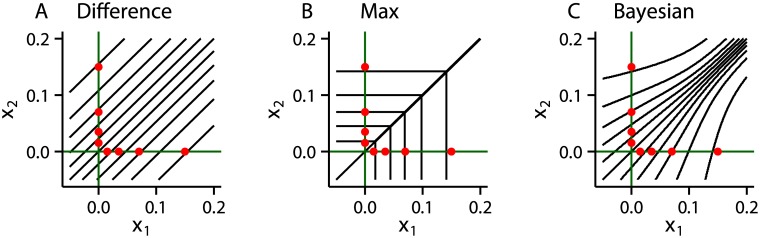
The mapping from stimulus-space to confidence induced by different models. The axes represent the two stimulus dimensions (cf. interval 1 and 2). The red dots represent the mean values of *x*_1_ and *x*_2_ for each stimulus. The black lines separate regions in stimulus space that map to a given confidence level. **A** Difference model. **B** Max model. **C** Bayesian model. The model parameters are the same as in Fig 8.

To understand this apparent continuum, we need to look at how the models map sensory data, defined by *x*_1_ and *x*_2_, onto confidence reports. We therefore plotted black contours dividing the regions of sensory-space (i.e. (*x*_1_, *x*_2_)-space) that map to different confidence levels (Fig 9). These plots highlight striking differences between the models. In particular, the Difference model has diagonal contours, whereas the Max model has contours that run horizontally, vertically or along the central diagonal at *x*_1_ = *x*_2_. In further contrast, the Bayesian model has curved contours with a shape somewhere between the Difference and Max models. In particular, for large values of *x*_1_ and *x*_2_, the contours are almost diagonal, as in the Difference model whereas for small values of *x*_1_ and *x*_2_, the contours are more horizontally or vertically aligned, as in the Max model.

To see how differences in the mapping from sensory-space to confidence reports translate into differences in the probability distribution over confidence reports, we consider the red dots, representing different target intervals and contrasts. For instance, a high-contrast target in interval 2 (*s* = 0.15), is represented by the uppermost red dot in each subplot. Importantly, red dots representing stimuli lie along the horizontal and vertical axes (green). The angle at which the contours cross these axes therefore becomes critically important. In particular, for the Difference model the contours pass diagonally through the axes, and therefore close to many red dots (representing stimuli), giving a relatively broad range of confidence levels for each stimulus type. In contrast, for the Max model, the contours pass perpendicularly through the axes, minimizing the number of red dots (representing stimuli) that each contour passes close to, giving a narrower range of confidence levels for each stimulus type. The contours of the Bayesian model pass through the axes at an angle between the extremes of the Difference and Max models—as expected, giving rise to a range of confidence levels between the extremes of the Difference and the Max model.

In principle, these differences might allow us to choose between models based only on visual inspection of *P*(*d*, *c*∣*i*, *s*, *m*, params). However, in practice, the distribution over decision and confidence reports, averaging over trial type, *p*_*d*, *c*_, is not constant, as we assumed above, but is far more complicated. This additional complexity makes it impossible to find the correct model by simple visual inspection. More powerful methods, like Bayesian model selection, are needed to pick out these differences.

## Discussion

We tested whether subjects’ confidence reports in a visual two-interval forced-choice task reflect heuristic or Bayes optimal computations. We assumed that subjects receive a two-dimensional sensory signal, **x**, and, based on that signal, make a decision (about which interval a target is in), and report their confidence in that decision. We also assumed that this process is mediated by intermediate variables: subjects transform those sensory signals into a continuous decision variable, *z* ^D^(**x**), compare this variable to a single threshold to make a decision, *d*, transform the sensory signals and the decision into a continuous confidence variable, *z* ^C^(**x**; *d*), and compare this variable to a set of thresholds to obtain a confidence level, *c*. We compared three possible ways of computing the confidence variable, *z* ^C^(**x**; *d*): the Difference model, which computes the difference between the sensory signals; the Max model, which uses only the sensory signal from the selected interval; and the Bayesian model, which computes the probability that a correct decision has been made. We used Bayesian model selection to directly compare these models. For the more standard, and perhaps more natural, design in which subjects first make a decision, and only then give a confidence rating (i.e. the two-response design), the Bayesian model emerged as the clear winner. However, for the less standard design, in which subjects make a decision and give a confidence rating simultaneously (i.e. the one-response design), the results were more ambiguous—our data indicated that around half of the subjects favoured the Bayesian model while the other half favoured the Max model.

One possible reason for the difference is that, in the one-response design, the computations underlying confidence reports were simplified so as not to interfere with the computation of the decision, as expected under theories of cognitive load (e.g. [21, 22]) and dual-task interference (e.g. [23, 24]). Alternatively, despite the instructions being the same, the two types of task design might simply promote qualitatively different computations, with the one-response design promoting a “first-order” judgement about the stimulus intensity, whereas the two-response design promotes a “second-order” judgement about the correctness of a decision which—perhaps critically—has already been made. Surprisingly, the commonly used Difference model was by far the least probable model in both task designs.

A caveat in any Bayesian model selection is that we cannot test all possible heuristic computations. However, given the results in Figs 8 and 9, it seems our three models range across the continuum of sensible models—though it is certainly possible that, perhaps, the best model (at least for the one-response data) sits somewhere between the Bayesian and the Max models. More generally, our results indicate that very subtle changes in a task can lead to large changes in the computations performed, and in particular whether subjects use Bayes optimal computations.

### Relation to other studies

Barthelmé & Mamassian (2009) [27] went part-way towards realizing the potential of using multidimensional stimuli. Subjects were asked to indicate which of two Gabor patches they would prefer to make an orientation judgement about. Interestingly, and in contrast to our results, they found that subjects were more likely to use a heuristic strategy (similar to the Max model) than a Bayes optimal strategy. However, there were three aspects of their study that make it potentially less relevant to the question of whether confidence reports reflects Bayes optimal computations. First, our model selection procedure is fully Bayesian, and therefore takes account of uncertainty in model predictions, whereas their procedure was not. In particular, under some circumstances a model will make strong predictions (e.g. “the subject must make this decision”), whereas under other circumstances, the model might make weaker predictions (e.g. “the subject is most likely to make this decision, but I’m not sure—they could also do other things”). Bayesian model selection takes into account the strength or weakness of a prediction. Second, in real life (and in our study), people tend to report confidence using verbal (e.g. “not sure” to “very” sure) or numerical (e.g. 1 to 10) scales. In contrast, in Barthelmé & Mamassian (2009) [27], subjects simply made a forced choice between two stimuli. Third, in their study, the Difference model made exactly the same predictions as the Bayes optimal model, making it impossible to distinguish these computations.

There are, of course, other approaches for addressing the question of whether the confidence variable is Bayes optimal. Barthelmé & Mamassian (2010) [28] showed that subject’s confidence variable can take into account two factors (contrast and crowding) that might lead to uncertainty—as opposed to using only one factor. Similarly, de Gardelle & Mamassian (2014) [29] showed that subjects were able to accurately compare the confidence variable across different classes of stimuli (in this case orientation discrimination versus spatial frequency discrimination). These studies provide some, albeit indirect, evidence that confidence reports might indeed reflect probability correct, in agreement with our work.

### Variability in confidence

Confidence reports have been observed to vary with a range of factors that we did not consider here. For example, people have been shown to be overconfident about the accuracy of their knowledge-based judgements, but underconfident about the accuracy of their perceptual judgements (see [30] for a review). People’s general level of confidence may also vary with social context. When groups of people resolve disagreement, the opinions expressed with higher confidence tend to carry more weight (e.g. [31]), so group members tend to increase their confidence to maximize their influence on the group decision [32, 33]. They may also adjust their confidence reports to indicate submission or dominance, or cut their losses if they should turn out to be wrong (e.g. [34]). Lastly, people’s confidence reports may vary with more general social factors such as profession, gender and culture: finance professionals are more confident than the average population (e.g. [8]); men are more confident than women (e.g. [35]); and people from Western cultures are more confident than people from East Asian cultures (e.g. [36]).

Our method allows us to think about the variability in confidence reports as having two dimensions. The first (perhaps more superficial) dimension relates to the average confidence level, or confidence distribution. We might imagine that this dimension is primarily modulated by social context, as described above. The second (perhaps deeper) dimension relates to the computations underlying confidence reports. In our data, there do indeed appear to be individual differences in how people generate their confidence reports, and very subtle changes to the task appear to affect this process. We might therefore expect shifts in how people generate their confidence reports for tasks of different complexity. For example, it is not straightforward to solve general-knowledge questions, such as “What is the capital of Brazil?”, using Bayesian inference. While one could in principle compute the probability that one’s answer is correct, the computational load may be so high that people resort to heuristic computations (e.g. using the population size of the reported city). Future research should seek to identify how confidence reports change between task domains and social contexts—in particular, whether such changes are mostly due to changes in the computation used to generate the confidence variable (cf., *z* ^C^(**x**; *d*)), or due to changes in the mapping of this variable onto some confidence scale.

### Two types of optimality

Many studies have asked whether confidence reports, and hence metacognitive ability, are optimal (see [37], for a review of measures of metacognitive ability). However, our work suggests that there are (at least) two kinds of optimality. First, the transformation of incoming data into an internal confidence variable (i.e. *z* ^C^(**x**; *d*)) could be optimal—that is, computed using Bayesian inference. Second, the mapping of the confidence variable onto some external scale of confidence could be optimal (i.e. *c*(*z* ^C^(**x**; *d*))), but this depends entirely on the details of the task at hand. For instance, without some incentive structure, there is no reason why subjects should opt for any particular mapping, as long as their mapping is monotonic (i.e. reported confidence increases strictly with their confidence variable). Importantly, it does not seem that subjects use an optimal mapping, as evidenced by the large amount of research on “poor calibration”—that is, the extent to which the reported probability of being correct matches the objective probability of being correct for a given decision problem (e.g. [30, 38]). Even when there is an incentive structure, subjects only improve their calibration and never reach perfection (e.g. [34, 39]). Future research should seek to identify why poor calibration arises, and how it can be corrected.

### Conclusions

We asked how people generate their confidence reports. Do they take a heuristic approach, and compute some reasonable, but ultimately arbitrary, function of the sensory input, or do they take a more principled approach, and compute the probability that they are correct using Bayesian inference? When subjects first made a decision and then reported their confidence in that decision, we found that their confidence reports overwhelmingly reflected the Bayesian strategy. However, when subjects simultaneously made a decision and reported confidence, we found the confidence reports of around half of the subjects were better explained by the Bayesian strategy, while the confidence reports of the other half of the subjects were better explained by a heuristic strategy.

## Supporting Information

S1 Table The best fitting parameters for the one-responses dataset. The first variable, *σ*, represents the subject’s noise level, and the second variable, *b*, represents their lapse rate. These parameters are sensible: *σ* is of the order of values used to generate a target Gabor patch, which ranges up to 0.15, and *b* is typically lower than 1%.
(PDF)

S2 Table As S1 Table, but for the two-responses dataset. (PDF)

S1 Fig The empirical and fitted distributions over signed confidence given the signed contrast for the one-response dataset. The lines show the fitted models, and the points show the data. Each row gives the complete responses for one subject. Each column gives the responses for a particular signed contrast value. The axis has been square-root transformed, in order to emphasize differences in low probabilities.
(EPS)

S2 Fig As S1 Fig, but for the two-responses dataset. (EPS)
