# Supplementary material for: Doubly Bayesian Analysis of Confidence in Perceptual Decision-Making
Source: PLoS Comput Biol. 2015 Oct 30;11(10):e1004519. doi: 10.1371/journal.pcbi.1004519 (PMC4627723; doi:10.1371/journal.pcbi.1004519)
Supplement: S1 Table — The first variable, σ, represents the subject’s noise level, and the second variable, b, represents their lapse rate. These parameters are sensible: σ is of the order of values used to generate a target Gabor patch, which ranges up to 0.15, and b is typically lower than 1%. (PDF) [file pcbi.1004519.s001.pdf]

Participant	σ	b	Model
1	0.087	0.016	Difference
2	0.057	0.063	Difference
3	0.067	0.021	Difference
4	0.072	0.033	Difference
5	0.074	0.007	Difference
6	0.087	0.001	Difference
7	0.075	0.003	Difference
8	0.091	0.011	Difference
9	0.067	0.026	Difference
10	0.159	0.003	Difference
11	0.068	0.003	Difference
12	0.061	0.015	Difference
13	0.091	0.004	Difference
14	0.050	0.004	Difference
15	0.128	0.004	Difference
1	0.099	0.004	Max
2	0.072	0.021	Max
3	0.076	0.006	Max
4	0.092	0.007	Max
5	0.086	0.005	Max
6	0.096	0.002	Max
7	0.089	0.001	Max
8	0.105	0.004	Max
9	0.082	0.015	Max
10	0.150	0.001	Max
11	0.080	0.002	Max
12	0.077	0.001	Max
13	0.112	0.001	Max
14	0.060	0.002	Max
15	0.144	0.002	Max
1	0.094	0.001	Bayesian
2	0.066	0.041	Bayesian
3	0.077	0.017	Bayesian
4	0.083	0.059	Bayesian
5	0.082	0.011	Bayesian
6	0.095	0.002	Bayesian
7	0.085	0.008	Bayesian
8	0.107	0.019	Bayesian
9	0.082	0.001	Bayesian
10	0.136	0.002	Bayesian
11	0.075	0.002	Bayesian
12	0.073	0.002	Bayesian
13	0.100	0.002	Bayesian
14	0.058	0.002	Bayesian
15	0.143	0.005	Bayesian
