# Supplementary material for: Doubly Bayesian Analysis of Confidence in Perceptual Decision-Making
Source: PLoS Comput Biol. 2015 Oct 30;11(10):e1004519. doi: 10.1371/journal.pcbi.1004519 (PMC4627723; doi:10.1371/journal.pcbi.1004519)
Supplement: S2 Table — (PDF) [file pcbi.1004519.s002.pdf]

Participant	σ	b	Model
1	0.059	0.002	Difference
2	0.080	0.116	Difference
3	0.053	0.052	Difference
4	0.060	0.008	Difference
5	0.072	0.007	Difference
6	0.075	0.025	Difference
7	0.079	0.004	Difference
8	0.141	0.002	Difference
9	0.092	0.003	Difference
10	0.078	0.016	Difference
11	0.062	0.052	Difference
1	0.073	0.001	Max
2	0.087	0.112	Max
3	0.056	0.068	Max
4	0.074	0.003	Max
5	0.081	0.005	Max
6	0.094	0.022	Max
7	0.090	0.001	Max
8	0.148	0.004	Max
9	0.106	0.018	Max
10	0.096	0.063	Max
11	0.072	0.074	Max
1	0.068	0.002	Bayesian
2	0.097	0.001	Bayesian
3	0.060	0.039	Bayesian
4	0.069	0.002	Bayesian
5	0.079	0.003	Bayesian
6	0.082	0.015	Bayesian
7	0.087	0.006	Bayesian
8	0.134	0.002	Bayesian
9	0.102	0.002	Bayesian
10	0.085	0.018	Bayesian
11	0.079	0.001	Bayesian
